# Simple ultraviolet microscope using off-the-shelf components for point-of-care diagnostics

**DOI:** 10.1371/journal.pone.0214090

**Published:** 2019-04-10

**Authors:** Cynthia Wong, Michal E. Pawlowski, Tomasz S. Tkaczyk

**Affiliations:** 1 Department of Bioengineering, Rice University, Houston, Texas, United States of America; 2 Department of Electrical and Computer Engineering, Rice University, Houston, Texas, United States of America; Universita degli Studi di Milano-Bicocca, ITALY

## Abstract

At the primary care setting, where there are often no or minimal laboratories, examinations often consist of self-testing and rapid diagnostics. Because of this, medical devices must be simple, robust, and easy to operate. To address these concerns, an alternate fluorescence microscope design uses ultraviolet (UV) excitation, since fluorescent dyes that are excitable in the visible region are also excitable by UV. This may allow for the removal of typical excitation, emission, and dichroic filters as optical components absorb UV wavelengths and UV is not detected by silicon based detectors. Additionally, UV has a very low penetration into samples, which may allow for controlling the depth of excitation, and thus the imaging volume. Based on these ideas, we developed a simple fluorescence microscope built completely from off-the-shelf components that uses UV to image fluorescently stained samples. The simple opto-mechanical design of the system may allow it to be more compact and easy to use, as well as decrease the overall cost of the diagnostic device. For biological validation, we imaged whole blood stained with acridine orange and performed a two-part white blood cell differential count.

## Introduction

At the primary care setting, such as in the developing world and underserved communities, there are often few laboratories and personnel to address the overall medical need; as a result, most examinations consist of self-testing and rapid diagnostics [1]. Because of this, medical devices used at these settings must be simple, robust, and easy to operate. Previously, low cost fluorescence microscopes have been designed for blood and enzyme-linked immunosorbent assay (ELISA) imaging and analysis [2–4]. Though these tested systems were successful at performing white blood cell (WBC) differential counting and bead-based bioassay tests, tight sample placement tolerances [2] and the need to change excitation and emission filters to image different channels [4] made the devices difficult to work with. Additionally, out of focus WBCs or beads from a deeper part of the sample contributed to background signal, and if not adjusted for correctly could potentially confound automated analysis [2–4].

To address these issues, an alternate fluorescence microscope design uses ultraviolet (UV) illumination as an excitation light source. The idea of UV as an excitation source was first discovered by Sir George G. Stokes in 1852, who observed that the mineral fluorspar emitted red light under illumination by UV wavelengths [5]. While there is little documentation on fluorescence in the short range UV, fluorescent dyes that are excitable in the visible region are also excitable by UV. This phenomenon was used to image fluorescently stained biological tissue samples for slide-free histology and pathology [6]. Though this system was not filter-less, the use of UV may allow for the removal of typical excitation, emission, and dichroic filters since most optical glasses absorb UV wavelengths (and thus act as natural filters) and UV is not normally detected by silicon based detectors, as the silicon semiconductor band-gap makes it sensitive in the visible and the near infrared part of the spectrum. In addition to the potential for filter-less imaging, UV has very low penetration into samples, which may allow for controlling imaging volume and reducing out of focus signals as only a small depth of the sample will be excited at any given time.

Based on these ideas, we developed a simple fluorescence microscope built completely from off-the-shelf components that uses UV as the illumination and can image any fluorescently stained sample, given that the fluorophore of interest can be excited by UV. By exploiting excitation in UV and through the proper selection of materials, the lenses and windows in the optical path may serve as natural filters, eliminating the need for swapping dedicated filters for the application of interest. The simple opto-mechanical design of the system may additionally allow it to be more compact, robust, and easy to use at the primary care setting, as well as decrease the overall cost of manufacturing the diagnostic device. To validate the ability of the system to analyze biological samples, we performed a two-part WBC differential count on whole blood stained with the fluorescent dye acridine orange (AO).

## Methods and materials

### Design of a fluorescence microscope using UV excitation

**Fig 1:**
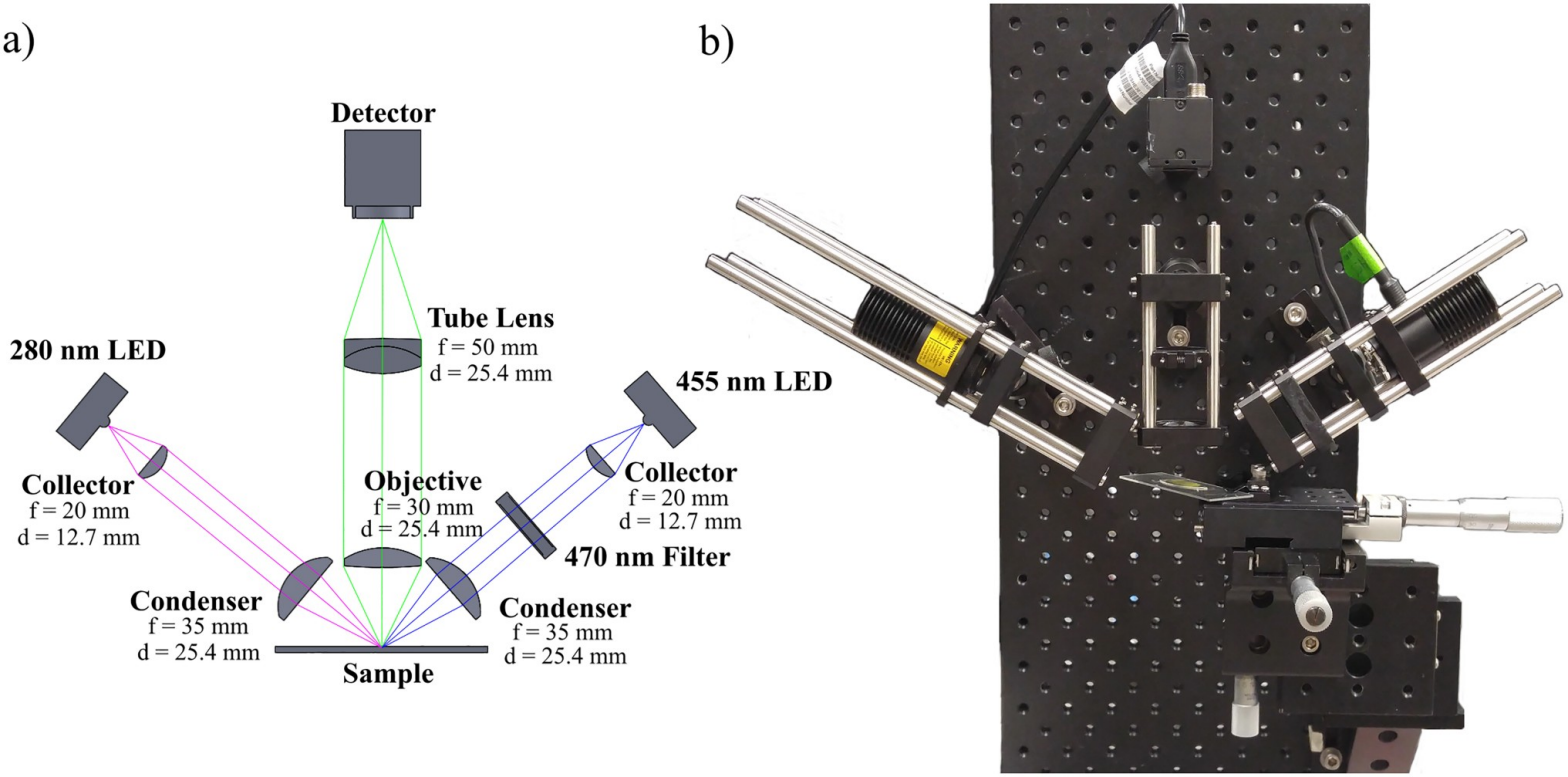
Optical schematic and image of the UV fluorescence microscope system. (a) Optical schematic of the designed UV microscope and (b) image of the fully assembled system. Samples were mounted on an xyz stage to enable focus adjustment and lateral scanning.

An optical schematic of the prototype fluorescence microscope using UV excitation is presented in Fig 1a and a photograph of the assembled system is shown in Fig 1b. The illumination system, aligned as critical illumination, consisted of two light emitting diodes (LED) at 280 nm (M280D2, Thorlabs, USA) and 455 nm (M455L3, Thorlabs, USA). These were chosen to allow for imaging with UV and standard fluorescence. A 20 mm focal length collector (LA4647 for the 280 nm LED and LA1074-B for the 455 nm LED, Thorlabs, USA) and a 35 mm focal length condenser lens (LA4052 for the 280 nm LED and LA1027-B for the 455 nm LED, Thorlabs, USA) were used to focus light on the sample.

The two illumination systems were aligned at an oblique angle to eliminate the need for a dichroic filter. No excitation or emission filters were needed for the 280 nm LED since the optical components (such as the objective and tube lens) should naturally absorb UV wavelengths, and the detector, a Point Grey Flea3 color camera (FL3-U3-88S2C-C, Point Grey Inc., Canada), was not sensitive to UV wavelengths as it had a silicon based detector tuned for the visible spectrum. Essentially, the optical components and the detector would act as the filter for the 280 nm illumination scheme. However, without excitation and emission filters in the 455 LED setup, images were saturated with the visible LED light and no emission signal was able to be imaged. Thus, a 470 nm excitation filter (FF01-470/28-25, Semrock, USA) and a 532 nm longpass emission filter (BLP01-532R-25, Semrock, USA) were needed to allow for standard fluorescence imaging with the visible spectrum LED. Samples were mounted on an xyz translation stage, where focus was adjusted using the z axis stage and lateral scanning of the sample was facilitated by movement of the x and y stages.

**Fig 2:**
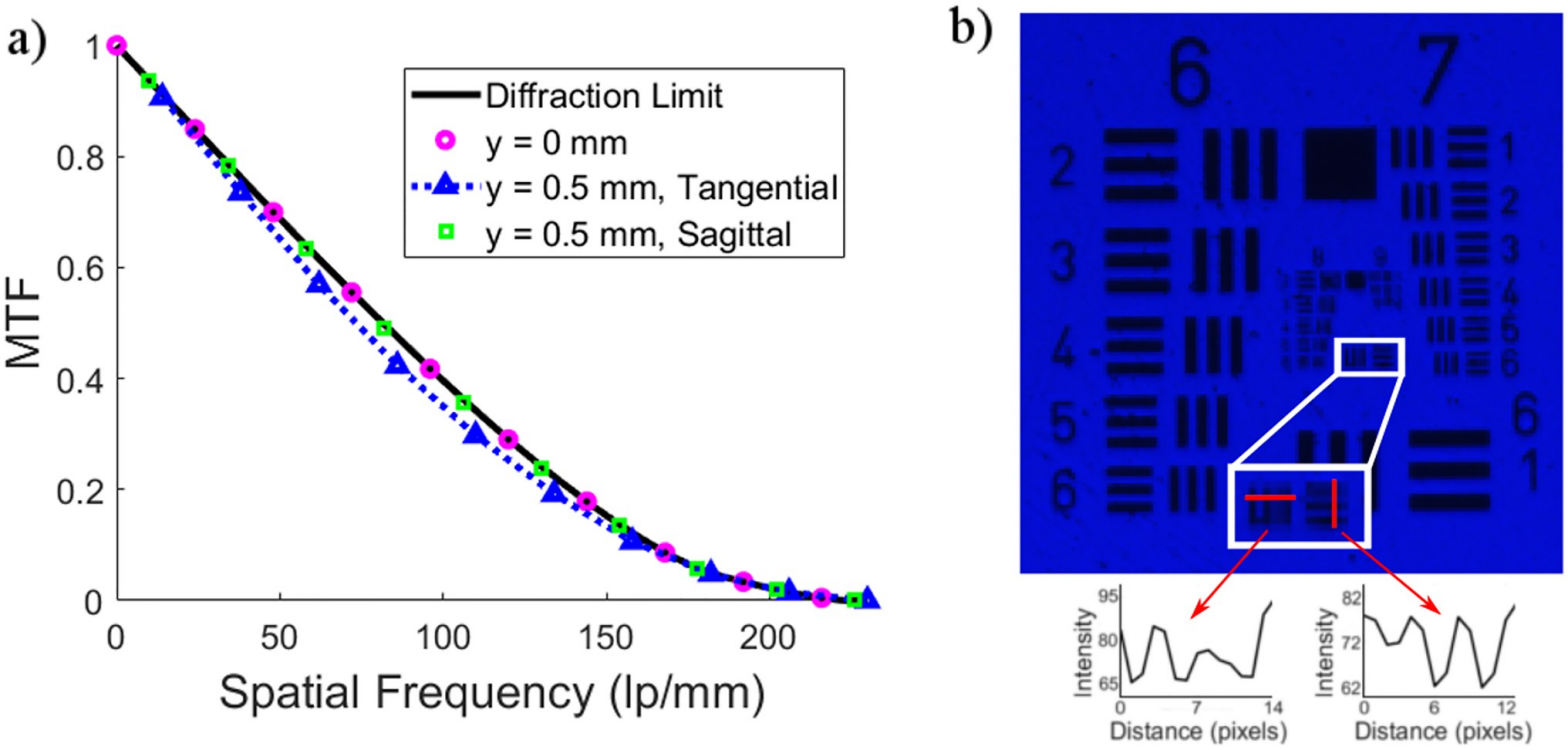
Performance of the assembled fluorescence microscope. (a) MTF plot calculated for the image plane in both the tangential and sagittal directions showing the nominal performance of the system. (b) Image taken with a 455 nm LED of a high resolution 1951 USAF target presenting the measured performance of the system. The smallest resolvable feature on the target was group 8 element 1 (shown enlarged and outlined in white), demonstrated by the intensity profiles through the vertical and horizontal elements indicated by the red lines and arrows. Resolution target image has been contrast enhanced for visualization purposes.

A summary of the optical design parameters for the UV fluorescence microscope can be seen in Table 1. The diameter of the field of view (FOV) was designed to be 1 mm to ensure that the system would be able to image at least 100 WBCs, which is necessary for statistically meaningful differential data [7]. To keep the system compact, short focal length lenses were considered for the objective, though the working distance needed to be at least 10 mm to allow light from the oblique angle illumination system to reach the sample. The numerical aperture (NA) was set to be 0.1 and the system had to be optimized for the two emission lines of AO (525 nm and 650 nm) to allow for imaging fluorescently stained WBCs, which have a minimum diameter of around 10 μm [8]. This meant that one of the lenses would need to perform chromatic correction (common lenses that did so included achromatic doublets). Additionally, due to the pixel size on the chosen detector (which had a chip size that was approximately 6 mm in width and 4 mm in height) and the desired FOV, magnification of the system needed to be between 1x and 4x. To ensure the simplicity of the system and to reduce cost, commercially available lenses were chosen to act as the objective and the tube lens. Based on these parameters and system requirements, Zemax (Radiant Vision Systems, USA) simulations were performed on pairs of lenses from the Edmund Optics and Thorlabs catalogs until the system gave diffraction limited performance (Strehl ratio of 0.8) at all field points. Ultimately, a pair of lenses were chosen that had the desired performance in an infinity-corrected fluorescence microscope configuration (Strehl ratio of 0.999 on axis and 0.940 at the edge of the field): a visible spectrum coated hybrid aspheric singlet with a 30 mm focal length (Part no. 66003, Edmund Optics, USA) and an aspherized achromatic doublet with a 50 mm effective focal length (Part no. 49665, Edmund Optics, USA). The working distance and image distance were optimized in Zemax and the infinity space allowed for the insertion of emission filters as needed for the 455 nm LED sub-system. The theoretical performance of the system is shown in the modulation transfer function (MTF) plot in Fig 2a.

**Table 1:** Summary of optical design parameters for the UV fluorescence microscope.

Object side FOV diameter [mm]	1.0
Objective working distance [mm]	26.0
Object side NA	0.10
Magnification	-1.67x
Curvature of the object surface [mm]	0.0
Curvature of the image surface [mm]	0.0
Design wavelength [nm]	525 and 650

After assembling the fluorescence microscope, performance of the system was verified using a high resolution 1951 United States Air Force (USAF) target (Stock No. 58–198, Edmund Optics, USA). A 455 nm LED was placed in trans-illumination mode to simplify initial testing of the aligned system (all other fluorescence imaging was performed with oblique angle illumination) and an image was taken, as seen in Fig 2b. The smallest resolvable element of the USAF target was that of group 8 element 1 (256 lp/mm), shown in the enlarged white outlined image outlined in Fig 2b. This limit was found according to the Rayleigh resolution limit, determined by taking an intensity cross-section through the vertical and horizontal elements indicated by the red lines and intensity plots below the resolution target image. Since the system was expected to work with fluorescence samples that did not need to resolve morphological details and WBCs have a minimum diameter of around 10 μm [8], the system performance was deemed sufficient for WBC analysis.

### Biological sample significance and preparation

The total number of WBCs, levels of agranulocytes and granulocytes, and the ratios of the different WBC types are important in determining whether a patient is infected with a bacterial or viral infection [9]. For example, patients presenting with high levels of granulocytes (granulocytosis) are more likely to have a bacterial infection and thus may need to be treated with antibiotics. Verifying the type of infection may be helpful in defining the proper treatment regimen for a patient, potentially improve therapy outcomes, and reduce overuse of antibiotics [10].

To find the levels and types of WBCs in a sample, WBCs may be fluorescently stained with acridine orange (AO), a metachromatic dye that binds to both DNA and RNA. AO fluoresce at 525 nm when bound to DNA and 650 nm when bound to RNA; thus, WBCs stained with AO fluoresce a mixture of red and green. The red-to-green (R/G) fluorescence ratio may then be used to identify WBCs and subsequently enable a two-part WBC differential count [11–13]. In all experiments presented in this paper, venous blood taken from healthy adult donors was stained with AO, where one milliliter of blood was prepared to have a final AO concentration of 10 μg/ml [14]. Ten μl of the AO stained blood was injected into a single chamber of the INCYTO C-Chip Disposable Hemacytometers, SKC (82030–468, VWR, USA). Samples were then imaged within three minutes of preparation with the UV excitation subsystem.

## Results

### Imaging of whole blood stained with AO

**Fig 3:**
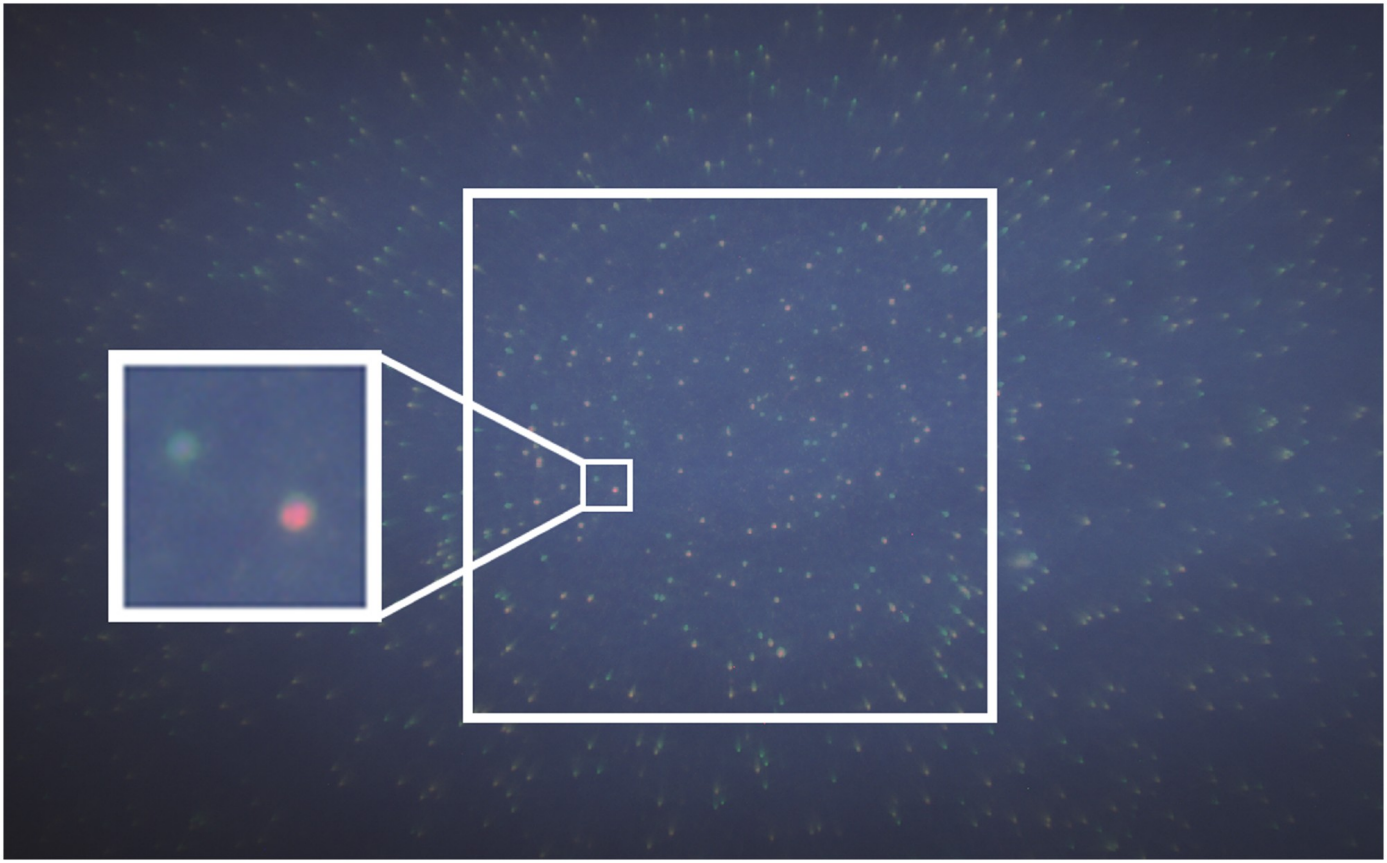
Fluorescence image of WBCs stained with AO. (a) Image taken using the 280 nm excitation source with an exposure time of 500 ms and detector gain of 0 dB without excitation or emission filters. The large, central white box represents the 1 mm x 1 mm FOV where WBCs are in focus. Insert image shows a pair of arbitrarily selected WBCs. (b) Image of the same FOV with an inexpensive absorption foil, which served as an emission filter, that only allowed the fluorescence signal from the WBCs. Color images were reconstructed from raw intensity values given by the Bayer mask of the camera.

Fig 3a shows an example fluorescence image of whole blood stained with AO as excited by the 280 nm LED. Color images were reconstructed from raw intensity values given by the Bayer mask of the camera to avoid potential inaccuracies from the color interpolation program typically used in the image capturing software. The large white box in the center shows the 1 mm x 1 mm FOV; within it, a pair of WBCs has been enlarged for visualization purposes, one being predominantly green and one with a mixture of green and red. The size of the FOV was chosen to ensure that the system would be able to image at least 100 WBCs, which is necessary for statistically meaningful differential data [7]. All image analysis was performed within this designed FOV. With the 280 nm LED, no filters were needed to clearly visualize the WBCs. Note that there was a blue background signal visible behind the WBCs, which is not typically seen in images such as in Fig 3b where an inexpensive absorption foil (#14 Medium Straw, Roscolux, USA) was used as an emission filter for comparison.

In an attempt to determine the cause of the blue background, tests were performed with a USB-650 Red Tide Spectrometer (OceanOptics, USA). These tests showed that the 280 nm LED appeared to bleed into the visible spectrum, ranging from 350 to 625 nm and with a strong peak around 425 nm. A 300 nm shortpass excitation filter (FF01-300/SP-25, Semrock, USA) was temporarily placed in the illumination path in an attempt to cut out the visible light leakage, as the shortpass filter would block any wavelength of light longer than 300 nm from passing through. However, measurements of the average background intensity with and without the filter revealed no significant difference in intensity. Though the objective is an optical polymer and autofluorescence was suspected, images taken of an empty sample cartridge revealed no observable signal on the detector. Additionally, images taken with unstained blood samples exhibited the same blue background when imaged under UV. Therefore, it was unlikely that the blue background signal originated from the 280 nm LED. Insertion of an inexpensive absorption foil, which served as an emission filter, did allow for a decrease in the background signal as seen in Fig 3b; however, analysis of WBCs from images without filters, presented in the following sections, show that a two-part differential count of whole blood was achievable despite this background signal.

### Two-part differential count of whole blood stained with AO

**Fig 4:**
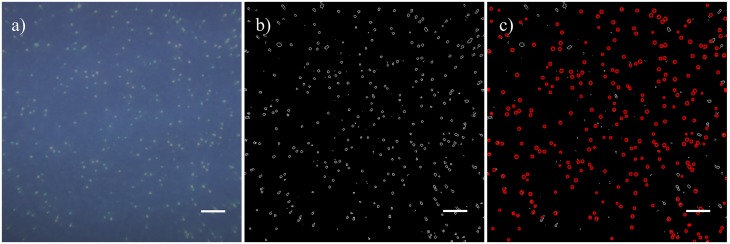
Segmenting WBCs from the FOV. (a) Image of AO stained whole blood taken under UV excitation in the 1 mm x 1 mm FOV, (b) WBCs after applying a Sobel filter, and (c) the segmented WBCs, displayed as the red circles, used for analysis based on the size criteria. Scale bars represents 100 μm.

**Fig 5:**
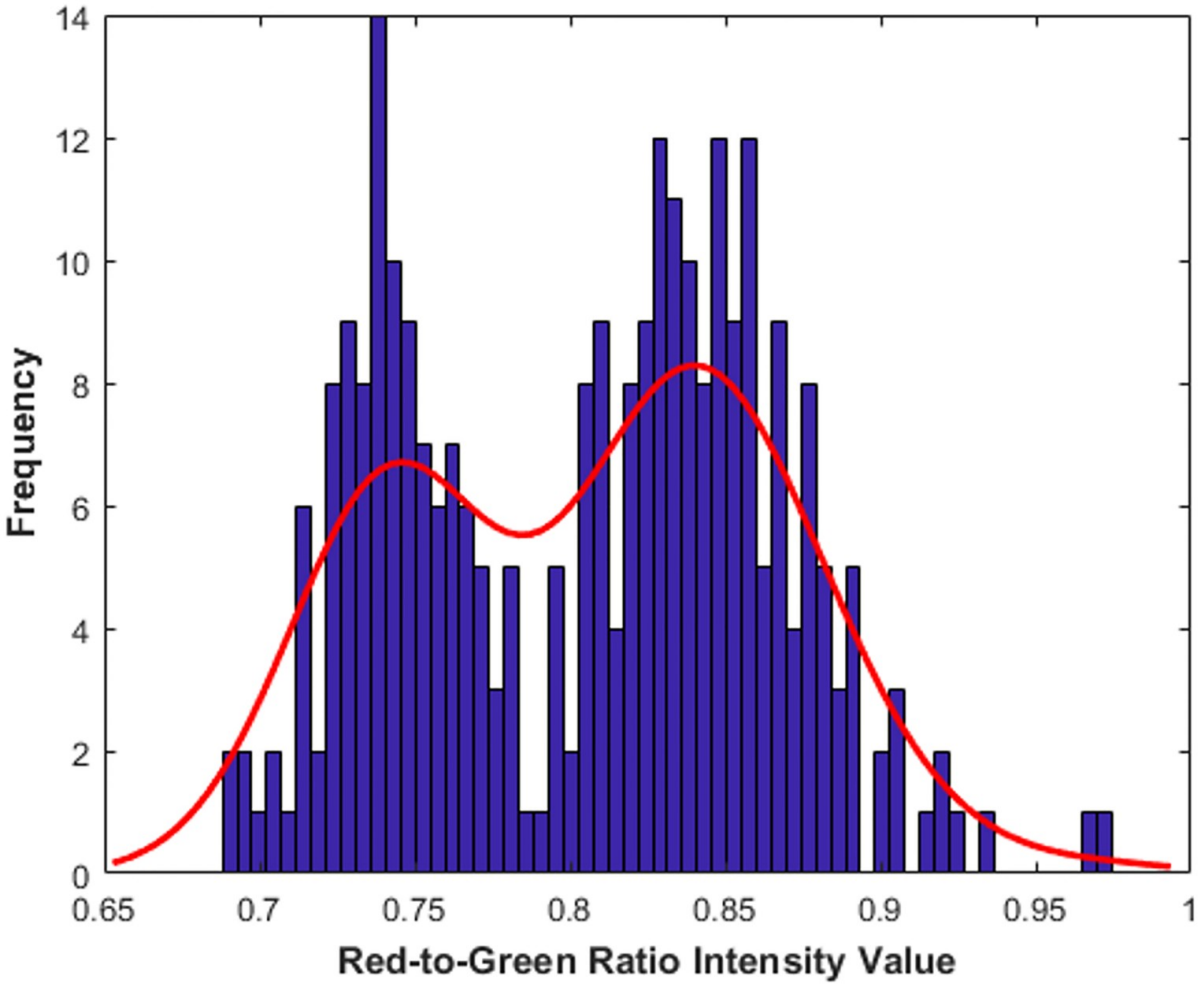
Example histogram of the R/G ratios in a single FOV. The red line shows the bimodal shape of the histogram, delineating what appears to be two distinct populations of WBCs (agranulocytes in the left population and granulocytes in the right population).

A two-part differential WBC count was performed for five separate blood samples, which were obtained from healthy volunteers who gave written informed consent under a protocol approved by the Rice University Institutional Review Board (620119). To segment and analyze the WBCs (illustrated in Figs 4 and 5), a custom automated program was written in MATLAB. In the first step of the image analysis process, the 1 mm x 1 mm FOV was cropped from the full image as shown in Fig 4a. Then, a Sobel filter was applied to the image to separate the circularly shaped WBCs from the background, the results of which are in Fig 4b. To exclude unwanted features, such as noise or overlapping WBCs, objects from the filtered image that did meet the experimentally established size criteria (between four and nine pixels in diameter) were eliminated. The remaining WBCs that were then used for analysis are shown as red circles in Fig 4c.

Once the WBCs were segmented, the R/G intensity ratio was calculated for each WBC in the FOV and a histogram of the R/G ratios was displayed. An example histogram for a single FOV may be seen in Fig 5, where the red line shows the overall bimodal shape of the histogram and indicates what appear to be two populations of WBCs.

WBCs that fell in the left population were counted as agranulocytes, while WBCs that fell in the right population were counted as granulocytes. Taking these counts and dividing by the total number of WBCs gave the percentage of agranulocytes and granulocytes for that sample. To calculate the concentration, the number of WBCs counted in the FOV was divided by the imaging volume, 0.02625 ml, which was found by multiplying the 1 mm x 1 mm FOV area with half of the depth of field for the 525 nm emission as focused on the front cartridge surface. Depth of field was calculated using the equation

$$DOF=\frac{\lambda n}{NA^{2}}$$ (1)

where λ was 525 nm, n was the index of refraction (in air), and NA was the numerical aperture of the system. Calculated values were validated against a commercial AcT Diff2 hematology analyzer (Beckman Coulter, USA). Although half the depth of field was used as an estimate for the image thickness in this system (and was thus an estimation of the imaging volume), volume can be verified experimentally for future devices. Note that the thickness of the image slice was smaller than the depth of the chamber, which was 100 μm.

**Fig 6:**
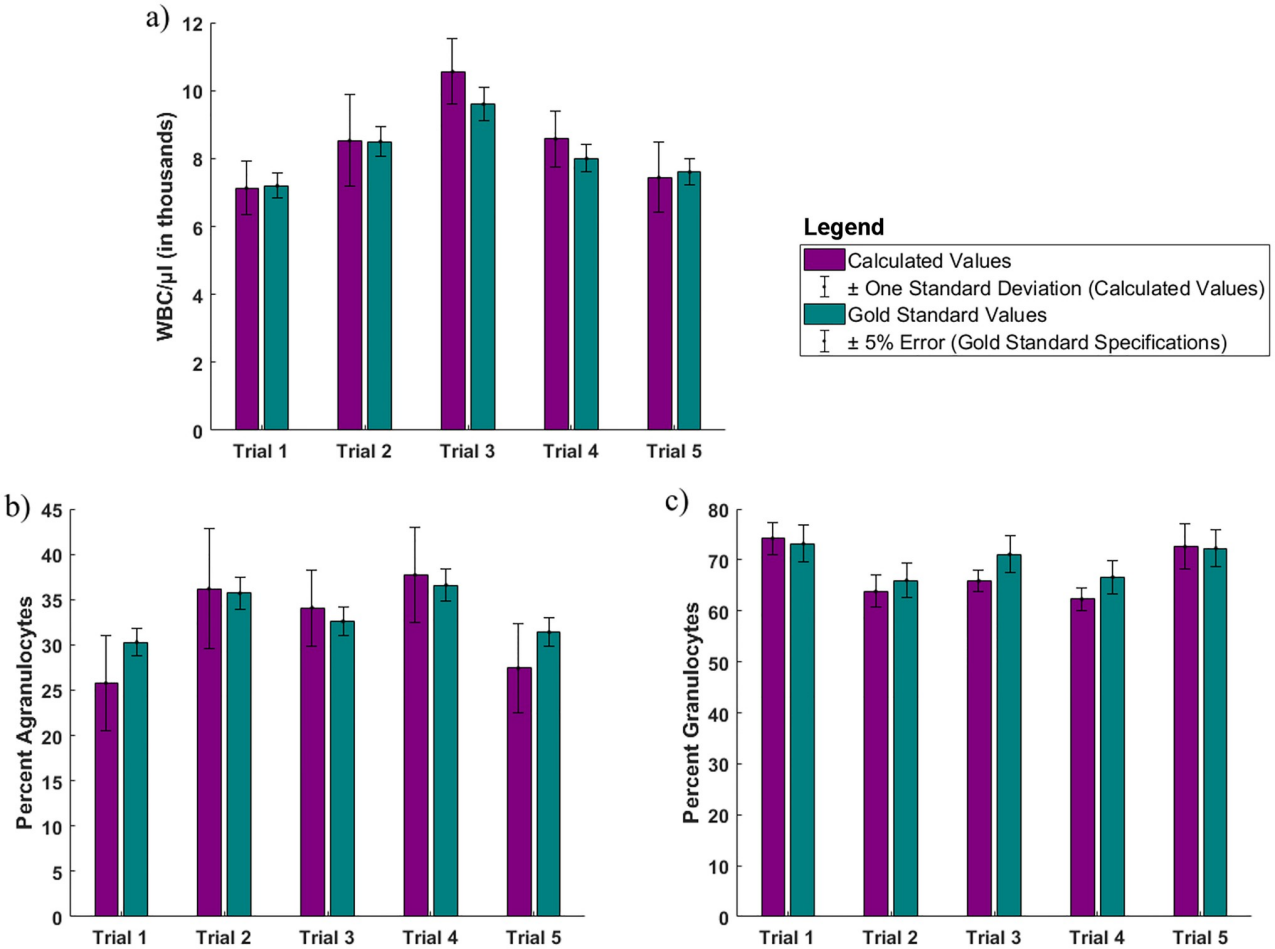
Comparison of calculated WBC values to commercial hematology analyzer reported values. Bar graphs show the (a) total WBC/μl, (b) percent agranulocytes, and (c) percent granulocytes for five separate venous draws. Purple bars on the left show the mean value of 12 FOVs as calculated by the algorithm (with error bars indicating ± one standard deviation) while turquoise bars on the right show the gold standard value as reported by the commercial hematology analyzer (with error bars indicating ± 5% error, given in the hematology analyzer specifications on accuracy [16]).

For each of the five separate venous draws performed, 12 FOVs were analyzed with a total imaging volume of 0.315 ml (as compared to 18 ml for the AcT Diff2 analyzer [15]). The WBC concentration, agranulocyte percentage, and granulocyte percentage values were calculated, and these values were compared to gold standard results reported by the commercial hematology analyzer. This data is summarized in Fig 6. Purple bars on the left show the mean value of 12 FOVs for each of the 5 venous draws as calculated by the algorithm (with error bars indicating ± one standard deviation) while turquoise bars on the right show the gold standard value as reported by the commercial hematology analyzer (with error bars indicating ± 5% error, given in the hematology analyzer specifications on accuracy [16]). All samples fell within ±15% of the true WBC concentration and percentage values. A range of ±15% from the gold standard result is a requirement for Clinical Laboratory Improvements Amendments (CLIA) waived devices.

## Discussions and conclusions

We designed and assembled a simple, filter-less fluorescent microscope that took advantage of the unique properties of fluorescent dyes under UV excitation. We also successfully developed automated post processing algorithms to segment WBCs from the designed FOV and performed a two-part WBC differential count. All of the tested samples fell within ±15% of the true WBC count. However, in the future (with additional system and algorithm optimizations) fewer FOVs may be necessary to obtain similar results to the gold standard. Further research and additional work will be needed to develop the system for three-part or five-part differentials, such as increasing both NA and magnification to allow for imaging the lymphocyte nuclei in more detail and more complex sorting algorithms to distinguish WBCs based on the additional morphological information.

The use of UV as an excitation source allows for a simpler fluorescent microscope in design, assembly, and usage. Typically, excitation, emission, and dichroic filters are needed to separate out the weak emission signals from the strong excitation signals, and are tailored for a specific application. This suggests that for every application the microscope would need different filter sets to properly image the sample of interest, which may be both expensive and inconvenient for streamlined use of the system. Optical glass components, such as lenses, naturally absorb UV wavelengths and silicon based detectors are not typically sensitive to electromagnetic waves from the UV range (though there are ways to make them sensitive [17]). By acting as the built-in “filters” of the system, UV allows for the removal of otherwise crucial optical components, simplifying the use and design of the fluorescent microscope. While the system was configured as an infinity corrected system (to allow for testing with standard fluorescence), further simplification may use a finite corrected setup, allowing for a single chromatically corrected lens/objective instead of a combination with an objective and a tube lens. Overall, the system can be built to be very compact and portable for use at the point of care, can be easily assembled through the use of off-the-shelf components, uses inexpensive test cartridges, and allows for further study on deployment and sustainability in the developing world. In addition, by removing the need for filters (which may be inconvenient when having to switch filters for imaging different samples), the microscope is more universal and easy to use for multiple applications. Future work will be needed to fully enclose and test the safety of the device as it involves the use of UV radiation.

The cost of the UV microscope can be broken down into two main components: the consumables (or the per test cost) and the base cost of the device. The cost of consumables has an important impact on sustainable use, especially in low income, developing world locations. Therefore, the system was designed to use accessible, low cost sample cartridges, which are priced at approximately $1 per test before any optimization (such as design customization, bulk purchasing, and more) and are compatible with the UV microscope. The chemistry of the cartridge and sample preparation are very basic and require only utilization of AO. This consumable price is much more affordable compared to a commercial system, such as the HemoCue WBC System (used at the point of care), where the cost of specially designed cartridges is approximately $3 per test [18] (which may be prohibitive for a developing country [19]). The second cost component of the UV microscope is that of the device itself, which costs approximately $750. This does not include the cost of the camera, as fluorescence microscopes are usually sold without a detector. The cost of the Flea3 camera used here is approximately $600, though it is possible to use other, less expensive detectors in the future. Typical filters cost at least $200–300 per filter [20], totaling at least $600 for a full filter set; if used, these filter may easily double the cost of the system. Though the base price of the device is comparable to the price of the HemoCue ($1099 [18]), the cost of the UV microscope is the retail cost before optimization. Improvements for mass production would help to drive down the price of the device. An additional advantage of the presented microscope is that it is not application specific and could be used for other fluorescence molecular detection applications. Note that standard microscope diagnostic tools allowing a broad range of applications are costlier. For example, a tuberculosis and malaria diagnostic system (such as a dedicated Zeiss Primo Star iLED, a fluorescence microscope designed to be used for infectious disease studies at the point of care) costs approximately $6000 [21].

Though the system was demonstrated for analyzing AO stained whole blood, other biological samples may be imaged with the system, provided that the fluorophore of interest can be excited by UV wavelengths. For example, enzyme-linked immunosorbent assays (ELISA) rely on fluorescent reporter and classifier beads to detect the presence of a substance such as an antigen. These beads fluoresce at different wavelengths and would normally need separate filters to image each type of bead individually. The use of UV excitation may eliminate the need for swapping filters, allowing one to take a single image and perform analysis on the bead signatures.
